# Supplementary material for: Overexpression of Potential Markers of Regulatory and Exhausted CD8+ T Cells in the Peripheral Blood Mononuclear Cells of Patients with B-Acute Lymphoblastic Leukemia
Source: Int J Mol Sci. 2023 Feb 24;24(5):4526. doi: 10.3390/ijms24054526 (PMC10003658; doi:10.3390/ijms24054526)
Supplement: Supplementary file 1 [file ijms-24-04526-s001.zip › Supplementary Figures.pdf]

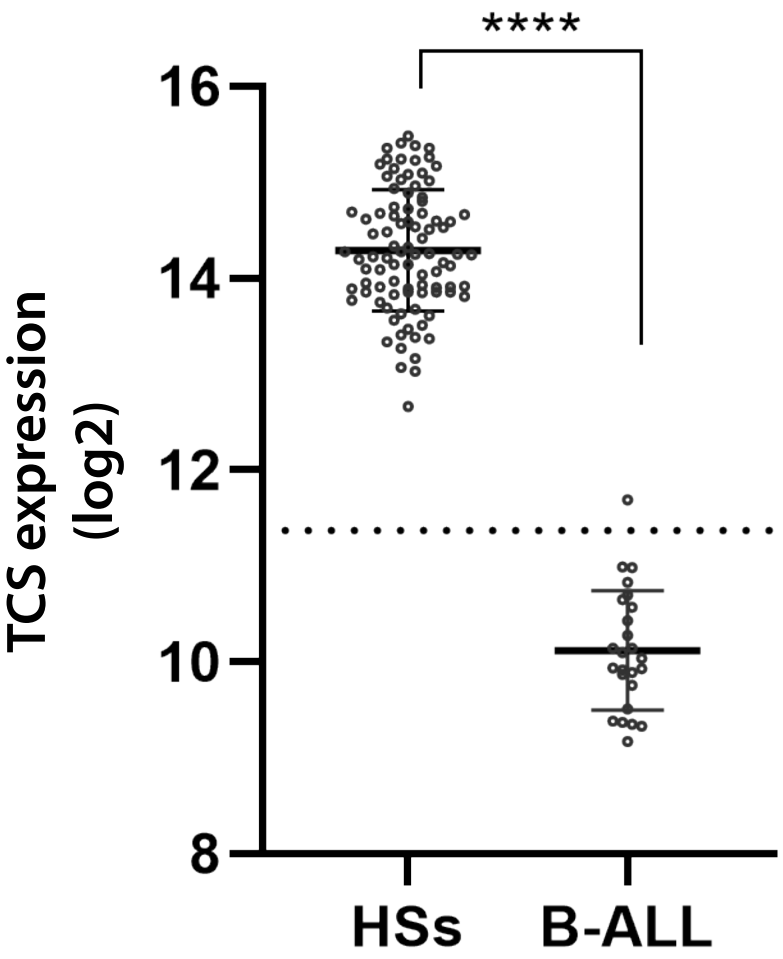

Figure S1. T cell signature expression in patients with B-ALL. The mean level of mRNA expression of T cell signature genes (TCS) in PBMC of patients with B-ALL is compared to that of healthy subjects (HSs). Mean ± standard deviation is also reported. The significant difference between B-ALL patients and HSs was evaluated using the t-test. **** $p<0.0001$

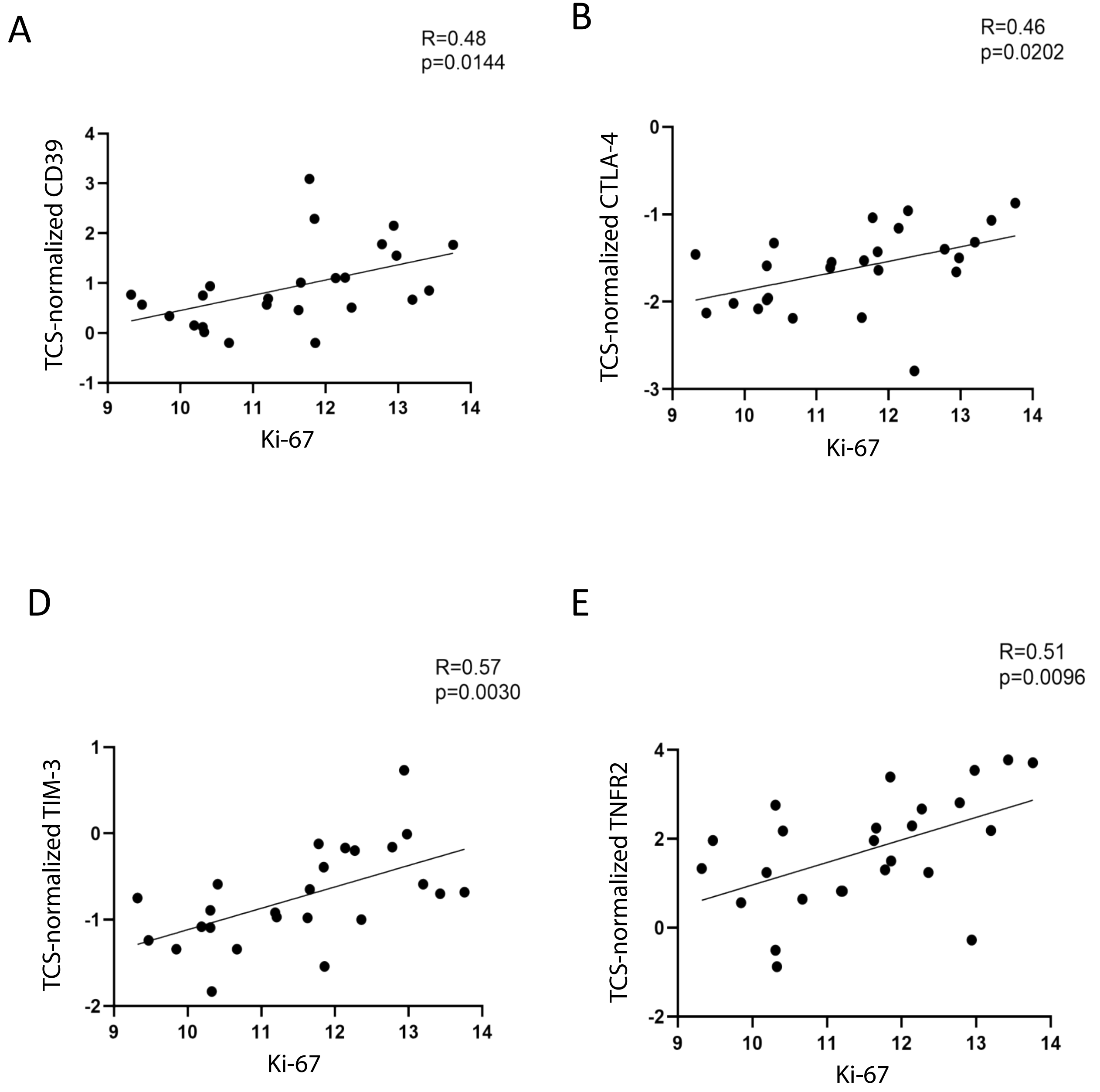

Figure S2. Correlation between Ki-67 and selected markers without cleaning the data from outliers. The correlation of mRNA expression of Ki-67 with normalized mRNA expression of CD39 (A), CTLA-4 (B), TIGIT (C), TIM-3 (D), and TNFR2 (E) is shown. Rho factor of correlation (R) and significance evaluated using the Pearson test are reported.
